# Orthorexic tendencies moderate the relationship between semi-vegetarianism and depressive symptoms

**DOI:** 10.1007/s40519-020-00901-y

**Published:** 2020-04-21

**Authors:** Johannes Baltasar Hessler-Kaufmann, Adrian Meule, Christina Holzapfel, Beate Brandl, Martin Greetfeld, Thomas Skurk, Sandra Schlegl, Hans Hauner, Ulrich Voderholzer

**Affiliations:** 1 Schoen Clinic Roseneck, Am Roseneck 6, 83209 Prien am Chiemsee, Germany; 2 Department of Psychiatry and Psychotherapy, University Hospital, LMU Munich, Munich, Germany; 3 Institute for Nutritional Medicine, School of Medicine, Technical University of Munich, Munich, Germany; 4 ZIEL-Institute for Food and Health, Technical University of Munich, Freising, Germany; 5 Chair of Nutritional Medicine, Else Kröner-Fresenius Center for Nutritional Medicine, Technical University of Munich, Freising, Germany; 6 Department of Psychiatry and Psychotherapy, University Hospital of Freiburg, Freiburg, Germany

**Keywords:** Vegetarianism, Depression, Orthorexia nervosa, Diet, Moderation

## Abstract

**Purpose:**

Vegetarianism and semi-vegetarianism (i.e., overly vegetarian diet with rare consumption of meat) have been repeatedly linked with depression. As the nature of this association is unclear, we explored whether orthorexic (i.e., pathologically healthful eating) tendencies and ecological/ethical motives to follow a vegetarian diet may moderate the relationship between (semi-)vegetarian diets and depressive symptoms.

**Methods:**

Five-hundred eleven adults (63.4% females; 71.2% omnivores, 19.2% semi-vegetarians, 9.6% vegetarians) completed the Patient Health Questionnaire (PHQ-9) questionnaire—measuring depressive symptoms—and the Düsseldorf Orthorexia Scale (DOS)—measuring orthorexic tendencies. Based on respective questions, participants were categorized as omnivores, semi-vegetarians, and vegetarians (including vegans) and were asked to indicate whether they chose their diet based on ecological/ethical motives. Moderation analyses were carried out with PROCESS.

**Results:**

Adjusted for age, sex, and body mass index, there was a statistically significant interaction effect between diet (omnivore vs. semi-vegetarianism vs. vegetarianism) and DOS scores when predicting PHQ depression scores. At low or medium DOS scores, diets did not differ in PHQ depression scores (all *p*s > 0.05). At high DOS scores, however, semi-vegetarians had higher PHQ depression scores than both omnivores (*p* = 0.002) and vegetarians (*p* < 0.001). The interaction between diet and ecological/ethical eating motives when predicting PHQ depression scores was not statistically significant (*p* = 0.41).

**Conclusion:**

Semi-vegetarians with strong orthorexic tendencies show more depressive symptoms than omnivores and vegetarians. The complex nature of the relationship between vegetarianism and depression requires further investigation.

**Level of evidence:**

III, case-control analytic studies.

## Introduction

Compared to omnivores, vegetarians and semi-vegetarians (i.e., occasional consumption of fish or meat, usually no red meat) have been found to show higher rates of depression, anxiety, and general mental distress [1–9]. Potential biological mechanisms assume depressive symptoms to result from low omega-3 fatty acid intake [10] or less protein-driven serotonin synthesis during cold seasons in (semi-)vegetarians [7]. These mechanisms, however, are incompatible with a range of findings. First, vegetarians experience lower rates of depression and other mental health issues than semi-vegetarians [2, 8, 9]. Second, Mediterranean and low inflammatory diets, which include little or no meat, were associated with a reduced incidence and prevalence of depression [11]. Third, self-report data suggested a change to vegetarianism after the onset of depression for two-thirds of a representative sample of the German general population [8].

The lack of sufficient biological explanations warrant the exploration of psychological variables that may reveal aspects of the association between (semi-)vegetarianism and depressive symptoms. The finding that orthorexic tendencies (i.e., obsession to eat healthy and maintenance of restrictive eating habits despite negative physiological and psychosocial consequences [12]) are more common in vegetarians [13–17] and relate to stronger depressive symptoms (for an overview see [18]), suggests that the motives to follow a vegetarian diet can offer insights into the circumstances under which vegetarianism relates to depression.

Next to health-related reasons, people usually choose to become vegetarians due to ecological and ethical reasons [19, 20]. Depression in vegetarians might, therefore, also result from an increased awareness of cruelty to animals in the meat industry and the detrimental environmental consequences of meat consumption [19], in a similar way as the confrontation with the issue of global warming may incite existential anxiety and depression [21].

So far, it has not been examined whether orthorexic tendencies and ecological/ethical motives influence the putative [16] association between (semi-)vegetarianism and depression. Therefore, we examined whether orthorexic tendencies or ecological/ethical motives moderate the association between diet (omnivorism vs. semi-vegetarianism vs. vegetarianism) and depressive symptoms.

## Methods

### Participants and procedures

Participants (*N* = 511, 63.4% female) were recruited from two studies at the Institute for Nutritional Medicine at the Technical University of Munich [22, 23]. Note that sample size is smaller for some variables [age: *N* = 503, body mass index (BMI): *N* = 507, PHQ depression score: *N* = 509; ecological and ethical eating motives: *N* = 510] because of missing data, but is larger than 500 for each variable reported in this article.

Participants were physically healthy, adult, and non-underweight volunteers from the general population. Detailed inclusion and exclusion criteria can be found in the respective studies [22, 23]. Mean age was 43.4 years (SD 18.1, range 18–84) and mean BMI was 25.2 kg/m^2^ (SD 4.7, range 17.6–51.2). Nine participants (1.8%) had completed lower school education [German: Hauptschule], 37 (7.2%) had completed middle school education [German: Realschule], 67 (13.1%) had completed higher school education [German: Gymnasium], 134 (26.2%) had completed vocational training, and 262 (51.3%) had a university degree (data missing for 2 participants, 0.4%). A set of questionnaires was completed either at the study center or was mailed to the participants. A reminder was sent to those who did not respond after one month. All participants gave written informed consent. The study was approved by the institutional review board of the University of Munich (#17-544) and the Technical University of Munich (#492/17S). Detailed descriptions of the study [18] and the psychometric measures [24] are reported elsewhere.

### Measures

The set of questionnaires included items on demographic and anthropometric data and several standardized scales.

Symptoms of ON were measured with the German Düsseldorf Orthorexia Scale (DOS [25]). The DOS’ 10 items inquire orthorexic eating behaviors (e.g., “I have certain nutrition rules that I adhere to.”) and associated emotions (e.g., “If I eat something I consider unhealthy, I feel really bad.”) and are rated on a four-point scale from “this does not apply to me” (1) to “this applies to me” (4). With total scores ranging from 10 to 40, values between 25 and 29 represent a risk of ON and values ≥ 30 are considered to represent ON. Internal reliability in the current study was good (McDonald’s *ω* = 0.86).

Depressive symptoms were measured with the German version of the 9-item Patient Health Questionnaire (PHQ-9 [26]). The nine items examine different aspects of depression and are rated on a 4-point scale ranging from 0 (“not at all”) to 3 (“nearly every day”). The sum score ranges from 0 to 27 with higher scores indicating more severe symptoms. Internal reliability in the current study was good (McDonald’s *ω* = 0.84).

Ecological and ethical eating motives were assessed with the items “I absolutely have to consider ecological aspects in my food choices” and “I absolutely have to consider ethical aspects in my food choices”, which were rated on a four-point scale from “not important at all” (1) to “very important” (4). As the two items had acceptable internal reliability (McDonald’s *ω* = 0.77), they were averaged for the present analyses.

We considered those persons as vegetarians (*n* = 49, 9.6% of the sample), who endorsed the statements “I eat vegetarian (no meat, no poultry, no fish)” or “I eat vegan (no products derived from animals)”. Vegans were included in the group of vegetarians as their group size was too small to allow for separate analyses. Those who negated these statements but endorsed the statement “I mostly eat vegetarian (no red meat but sometimes poultry and/or fish)” were considered as semi-vegetarians (*n* = 98, 19.2%). All others were considered as omnivores (*n* = 364, 71.2%).

### Statistical analyses

Omnivores, vegetarians, and semi-vegetarians were compared regarding age, BMI, PHQ depression scores, DOS scores, and ecological/ethical eating motives scores with analyses of variance. Significant group differences were followed up with independent *t* tests. Sex differences between groups were examined with *χ*^2^-tests. Associations between all study variables were evaluated with Pearson’s correlation coefficients. To examine whether orthorexic tendencies and ecological/ethical eating motives moderated the relationship between diet and depressive symptoms, two moderation models based on linear regression analyses were calculated with PROCESS version 3.4 [27, 28]. Specifically, group (omnivore, semi-vegetarian, vegetarian) was entered as a multicategorical independent variable, PHQ depression scores were entered as the dependent variable, and either DOS scores or ecological/ethical eating motives scores were entered as moderator variable. In both models, age, sex, and BMI were entered as covariates. *p* values of < 0.05 were considered to represent statistical significance.

## Results

### Group differences

Vegetarians, semi-vegetarians, and omnivores significantly differed in age (*F*_(2,500)_ = 10.2, *p* < 0.001), sex (*χ*^2^_(2)_ = 14.6, *p* = 0.001), BMI (*F*_(2,504)_ = 14.0, *p* < 0.001), PHQ depression scores (*F*_(2,506)_ = 3.5, *p* = 0.03), DOS scores (*F*_(2,508)_ = 8.3, *p* < 0.001), and ecological/ethical eating motives scores (*F*_(2,507)_ = 46.0, *p* < 0.001). Semi-vegetarians were younger, more likely to be female, had lower BMI, and had higher PHQ depression and ecological/ethical eating motives scores than omnivores (all *p*s < 0.05), but did not differ in DOS scores (*t*_(460)_ = 1.4, *p* = 0.15; Table 1). Vegetarians were younger, more likely to be female, had lower BMI, and had higher DOS and ecological/ethical eating motives scores than omnivores (all *p*s < 0.01), but did not differ in PHQ depression scores (*t*_(409)_ = 0.8, *p* = 0.43; Table 1). Vegetarians were also younger and had higher DOS and ecological/ethical eating motives scores than semi-vegetarians (all *p*s < 0.03), but did not differ in sex distribution, BMI, and PHQ depression scores (all *p*s > 0.32; Table 1).

**Table 1:** Descriptive statistics of the study sample and correlations between study variables

	Omnivores (*n* = 364)	Semi-vegetarians (*n* = 98)	Vegetarians (*n* = 49)	1	2	3	4	5	6
1. Age (years)	*M* = 45.3 (SD = 18.2)	*M* = 41.1 (SD = 18.1)	*M* = 33.8 (SD = 13.0)	–	0.15*	0.35*	− 0.09*	− 0.15*	− 0.16*
2. Sex (0 = female, 1 = male)	*n* = 212 female (58.2%)	*n* = 74 female (75.5%)	*n* = 38 female (77.6%)		–	0.15*	− 0.15*	− 0.10*	− 0.12*
3. Body mass index (kg/m^2^)	*M* = 25.9 (SD = 4.9)	*M* = 23.3 (SD = 3.9)	*M* = 23.8 (SD = 3.9)			–	0.15*	0.06	− 0.12*
4. Patient Health Questionnaire depression scores	*M* = 4.1 (SD = 3.6)	*M* = 5.2 (SD = 4.3)	*M* = 4.5 (SD = 3.2)				–	0.37*	0.01
5. Düsseldorf Orthorexia Scale scores	*M* = 16.0 (SD = 4.6)	*M* = 16.8 (SD = 5.3)	*M* = 19.0 (SD = 5.0)					–	0.15*
6. Ecological/ethical eating motives	*M* = 2.3 (SD = 0.7)	*M* = 2.7 (SD = 0.6)	*M* = 3.2 (SD = 0.6)						–

*M* mean, *SD* standard deviation

*p < 0.05

### Correlations between variables

Almost all study variables were weakly correlated with each other (Table 1). The DOS scores and ecological/ethical eating motives scores were weakly, positively correlated with each other, but only DOS scores were moderately, positively correlated with PHQ depression scores.

### Moderation analyses

**Fig. 1:**
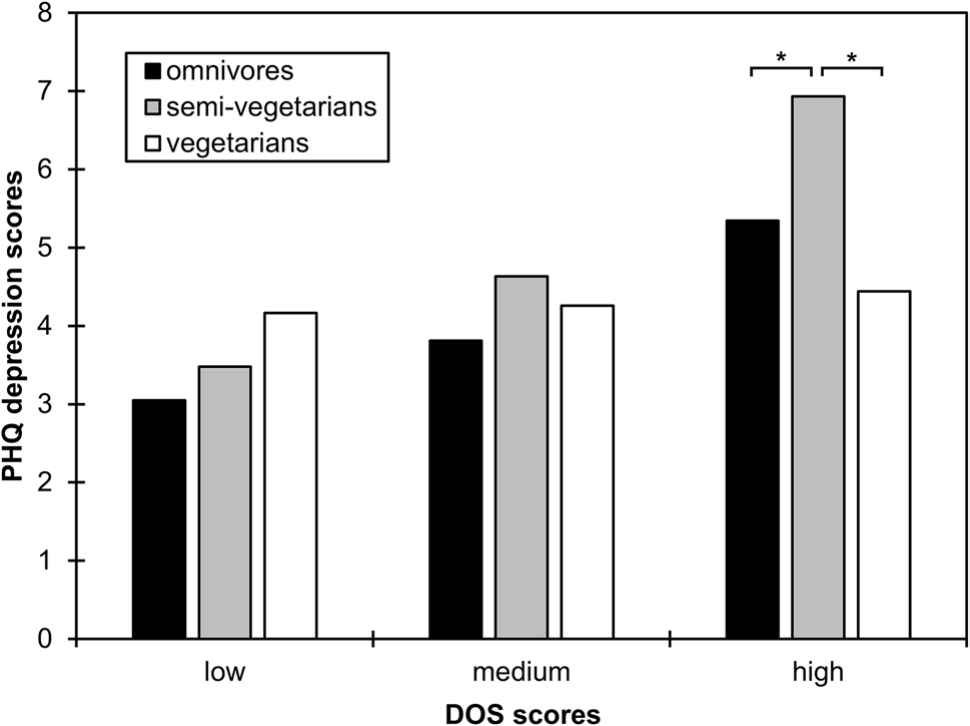
Group means of Patient Health Questionnaire (PHQ) depression scores as a function of scores on the Düsseldorf Orthorexia Scale (DOS). Low, medium, and high DOS scores represent the 16th, 50th, and 84th percentiles (which were scores of 12, 15, and 21 in the current study). Note that this does not mean that participants were categorized into groups based on DOS scores—the differentiation between low, medium, and high scores serves the purpose of probing the interactive effect between dietary group and DOS scores on PHQ depression scores. Asterisks indicate statistically significant differences at *p* < 0.05

As age, sex, and BMI differed between groups, these variables were included as covariates in the moderation models. There was a statistically significant interaction effect between group and DOS scores when predicting PHQ depression scores (*R*^2^ change = 0.02, *F*_(2,488)_ = 4.8, *p* = 0.01). At low or medium DOS scores, groups did not differ in PHQ depression scores (all *p*s > 0.05). At high DOS scores, semi-vegetarians had higher PHQ depression scores than both omnivores (*b* = 1.6, SE = 0.5, *t* = 3.1, *p* = 0.002) and vegetarians (*b* = 2.5, SE = 0.7, *t* = 3.7, *p* < 0.001; Fig. 1). The interaction between group and ecological/ethical eating motives when predicting PHQ depression scores was not significant (*R*^2^ change = 0.003, *F*_(2,487)_ = 0.9, *p* = 0.41), that is, ecological/ethical eating motives did not moderate the effect of group on PHQ depression scores.

## Discussion

In a sample from the general population, we found that orthorexic tendencies moderated the association between diet and depressive symptoms. In those persons with strong orthorexic tendencies, only those with a semi-vegetarian diet showed stronger depressive symptoms. Both a vegetarian diet (e.g., [3]) and orthorexic tendencies [18] have been individually linked to depressive symptoms. The moderating effect in our analyses indicates that these factors do not only contribute to depressive symptoms additively but interactively. That is if both factors are combined (i.e., someone eats partially vegetarian and exhibits strong orthorexic tendencies), depressive symptoms are elevated the most (Fig. 1). Furthermore, full vegetarians had stronger orthorexic tendencies but less depressive symptoms in our sample. Thus, it seems that at least for a subgroup of persons, neither vegetarianism nor orthorexic tendencies alone can fully explain depressive symptoms. Importantly, only very few participants in the current study showed DOS scores in ranges that are considered pathological. For the participants with moderate or low DOS scores, these values may, therefore, represent variations of actually healthful eating.

It could be speculated that persons who have high or even pathological health-related motives for their vegetarian diet but fail to follow this diet (i.e., they drift into a semi-vegetarian diet) are subject to the dissonance between their ideals and their behavior, which may then contribute to increased depressive symptoms. This is in line with the finding that health-driven vegetarians more often violate their dietary rules than morally driven vegetarians [29, 30]. In our sample, vegetarians had stronger orthorexic tendencies, which may relate to an ability to maintain their diet and derive a sense of achievement from that. Importantly, these hypotheses are of preliminary nature and require further investigation.

While ecological/ethical aspects seem to be the predominant motivation for adopting a vegetarian diet [19, 20], they did not moderate the association between diet and depressive symptoms in our sample. Again, vegetarians had the strongest ecological/ethical motives and semi-vegetarians showed stronger ecological/ethical motives than omnivores, yet, they did not relate to depressive symptoms in any dietary group. This finding indicates that the increased confrontation with detrimental effects of meat consumption on the environment and animals does not show the hypothesized negative effects on mood.

Our findings further confirmed previous results suggesting that semi-vegetarians show more pronounced depressive symptoms than vegetarians and omnivores [2, 8, 9]. However, depressive symptoms did not differ between vegetarians and omnivores, which does not confirm the majority of studies reporting better mental health in omnivores compared to vegetarians [1–9]. This finding instead emphasizes an equivocality in the literature, with some studies reporting better mental health in vegetarians compared to omnivores [10, 31, 32].

Together with vegans, vegetarians seem to show the strongest orthorexic tendencies, followed by semi-vegetarians and omnivores [13], which was confirmed by our results. While a vegan diet has been linked with predominant health-related motives [33], the vegetarians also showed stronger ecological/ethical motives in our sample, followed by semi-vegetarians and omnivores. It has been suggested that semi-vegetarians restrict their eating for the purpose of weight control, while vegetarians restrict their eating based on ethical concerns [34]. While eating according to one’s ethical standard may increase well-being [19], restrictive eating to control weight rather resembles symptoms of eating disorders, which are closely linked to depressive disorders [35–37]. Hence, it is possible that the semi-vegetarians with high DOS scores also showed the strongest depressive symptoms because they chose their restrictive eating style to control weight. More insight into the differential motives to become vegetarian or vegan and differences to semi-vegetarians is needed [19].

Motivations and reasons for becoming and staying vegetarian are multifold, interacting, and pertain to personal, social, and moral aspects [19], all of which may act as protecting or risk factors in the relationship between diet and mental health. Our study emphasizes that examining the motivation to adopt a certain diet may further illuminate these occult associations. Yet, our findings also show that the relationship between a vegetarian diet and depression is complex and suggest that there are additional factors that increase or decrease the likelihood that a (semi-)vegetarian diet is linked to depression.

Interpretation of the current results is limited by our sample, which was derived from studies on health and nutrition. The study sample is not representative and might be biased by including a substantial proportion of persons with either specific interests in nutrition or related health outcomes. Further, we had no means to assess the exact number of non-respondents and the reasons for their non-response as well as any differences between those who returned the questionnaire and those who did not, which may have resulted in a selection bias. Finally, we did not measure the participants’ diets through objective methods but by self-report, which precludes statements about the validity of the dietary groups.

## Conclusions

Semi-vegetarians with strong orthorexic tendencies showed stronger depressive symptoms than vegetarians and omnivores. A vegetarian diet per se does not seem to be related to higher depressive symptoms.

### What is already known on this subject?

Vegetarianism and semi-vegetarianism are associated with higher rates of depressive symptoms, while proposed explanations are insufficient.

### What this study adds?

Semi-vegetarianism is only associated with depressive symptoms in persons who also report higher orthorexic tendencies.
